# Tracheal Aspirate Metagenomics Reveals Association of Antibiotic Resistance with Nonpulmonary Sepsis Mortality

**DOI:** 10.1165/rcmb.2024-0192LE

**Published:** 2025-02-01

**Authors:** Héctor Rodríguez-Pérez, Laura Ciuffreda, Tamara Hernández-Beeftink, Beatriz Guillen-Guio, David Domínguez, Almudena Corrales, Elena Espinosa, Julia Alcoba-Florez, Jose M. Lorenzo-Salazar, Rafaela González-Montelongo, Jesús Villar, Carlos Flores

**Affiliations:** ^1^ Hospital Universitario Ntra. Sra. de Candelaria Santa Cruz de Tenerife, Spain; ^2^ Instituto de Investigación Sanitaria de Canarias Santa Cruz de Tenerife, Spain; ^3^ University of Leicester Leicester, United Kingdom; ^4^ NIHR Leicester Biomedical Research Centre Leicester, United Kingdom; ^5^ CIBER de Enfermedades Respiratorias-Instituto de Salud Carlos III Madrid, Spain; ^6^ Instituto Tecnologico y de Energias Renovables Santa Cruz de Tenerife, Spain; ^7^ Hospital Universitario de Gran Canaria Dr. Negrin Las Palmas de Gran Canaria, Spain; ^8^ St. Michael’s Hospital Toronto, Ontario, Canada; ^9^ Universidad Fernando de Pessoa Canarias Las Palmas de Gran Canaria, Spain

*To the Editor*:

Sepsis is a severe and life-threatening complication of infection characterized by a systemic inflammatory response and organ dysfunction (1). Despite significant advances in medical care, sepsis leads the causes of mortality in the ICU and remains a major cause of morbidity among survivors (2). The pathogenesis of sepsis is complex and multifactorial, involving interactions between host immune responses and the microbiome (3). Our previous studies in mechanically ventilated ICU patients with nonpulmonary sepsis revealed that pulmonary dysbiosis within 8 hours from sepsis diagnosis was associated with ICU mortality (4). These observations offer promising options that could be technologically adapted to rapidly identify those patients at higher risk of death (5). However, they relied on 16S rRNA gene sequencing experiments, which impedes deep characterization of the bacterial communities that could help to further substantiate the association. To further discern the association of lung dysbiosis with ICU mortality among patients with sepsis, here we used metagenomic sequencing for improved characterization of the bacterial lung communities and for adding support to the possible enrichment of antimicrobial resistances (AMRs) among the deceased patients. Some of the results of these studies have been previously reported in the form of a preprint (medRxiv, 10 Apr 2024, https://doi.org/10.1101/2024.04.08.24305484).

Tracheal aspirates from 32 mechanically ventilated patients (25 survivors, 7 deceased) admitted to the medical-surgical ICU within the first 8 hours from sepsis diagnosis (1) between January 2015 and January 2019 were included in the study (Table 1). Details for statistical power calculations of the cohort are detailed elsewhere (4), supporting that with just 24 patients we achieved >80% power to detect the observed differences at *P* < 0.05. Patients included in the study had a diagnosis of acute abdomen and constitute a subset of those with sufficient DNA material from aspirates whose clinical characteristics have been described elsewhere (4, 5). DNA sequencing libraries were prepared using the Nextera XT Library Prep Kit (Illumina Inc.), and sequencing was performed using a HiSeq 4000 system (Illumina Inc.). Metagenomic data were analyzed using an in-house bioinformatic pipeline. A logistic regression model was used to assess the association between antimicrobial resistance genes (ARGs) and ICU mortality. Additional details are provided in the data supplement.

**Table 1.** Demographic and Clinical Variables from Patients and Results from the Sensitivity Analysis of the Model Assessing the Association of the Abundance of Antimicrobial Resistances with ICU Mortality among Patients with Nonpulmonary Sepsis

	All Samples (*N* = 32)	Survivors (*n* = 25)	Deceased (*n* = 7)	*P* Value*	Adjusted Model	
					OR (95% CI)	*P* Value^†^
Sex, male	23 (71.87)	19 (76)	4 (57.14)	0.61	1.19 (1.03–1.41)	0.025
Age, yr	67.4 ± 11.36	66.24 ± 12.31	71.57 ± 5.88	0.29	1.18 (1.02–1.40)	0.035
APACHE II	25 (19–28)	22.5 (17.5–26.75)	27 (25–30.5)	0.06	1.27 (1.05–1.67)	0.031
SOFA	10 (8–11)	9 (8–11)	11 (10.5–11.5)	0.15	1.30 (1.07–1.70)	0.017
SAPS II	60 (54–69)	60 (51.5–69.5)	63.5 (58.5–68.5)	0.53	1.26 (1.03–1.68)	0.041
ARDS	5 (15.62)	5 (20)	0 (0)	0.48	1.30 (1.08–1.69)	0.012
Smoker	5 (15.62)	3 (12)	2 (28.57)	0.63	1.20 (1.04–1.43)	0.018
Previous severe infections^‡^	4 (12.5)	4 (16)	0 (0)	0.62	1.53 (1.15–3.46)	0.062
Infection source					1.18 (1.03–1.40)	0.028
Abdominal and gastrointestinal tract	27 (84.37)	20 (80)	7 (100)	0.48	1.14 (1.03–1.36)	0.050
Genitourinary system	3 (9.37)	3 (12)	0 (0)	0.81	1.15 (1.03–1.37)	0.045
Bones and soft tissues	2 (6.25)	2 (8)	0 (0)	1	1.15 (1.03–1.37)	0.042
Isolated pathogen					1.19 (1.03–1.41)	0.024
Gram-positive bacteria	6 (18.75)	4 (16)	2 (28.57)	0.83	1.16 (1.04–1.39)	0.032
Gram-negative bacteria	10 (31.25)	8 (32)	2 (28.57)	1	1.19 (1.05–1.49)	0.037
Gram-positive and gram-negative	4 (12.5)	3 (12)	1 (14.29)	1	1.17 (1.04–1.39)	0.031
Polymicrobial	8 (25)	6 (24)	2 (28.57)	1	1.16 (1.04–1.39)	0.038
Antibiotic treatment^§^	26 (81.25)	19 (76)	7 (100)	0.46	1.16 (1.01–1.37)	0.046
Multiorgan dysfunction^‖^	29 (90.62)	22 (88)	7 (100)	0.81	1.18 (1.02–1.39)	0.029
Comorbidities^¶^	14 (43.75)	10 (40)	4 (57.14)	0.7	1.18 (1.02–1.40)	0.030
Cancer	5 (15.62)	2 (8)	3 (42.86)	0.09	1.22 (1.05–1.50)	0.019
Arterial hypertension	16 (50)	12 (48)	4 (57.14)	1	1.19 (1.03–1.41)	0.024
Bicarbonate levels	42.63 ± 7.54	42.35 ± 6.2	43.57 ± 11.53	1	1.33 (1.10–1.77)	0.011
Hospital days	27.5 (26–36)	29 (17–58)	18 (14.5–27)	0.11	1.47 (1.13–2.33)	0.026
Hospital days ICU	11 (4.75–17.5)	9 (4–15)	16 (10–25)	0.26	1.19 (1.03–1.41)	0.020

*Definition of abbreviations*: APACHE II = Acute Physiology and Chronic Health Evaluation II at inclusion; ARDS = acute respiratory distress syndrome; CI = confidence interval; OR = odds ratio; SAPS II = Simplified Acute Physiology Score II; SOFA = Sequential Organ Failure Assessment.

Data are shown as counts (%), mean ± SD, or median (P_25_–P_75_; interquartile range). None of the variables had missing data.

* Comparisons between survivors and deceased. Age, severity scores, bicarbonate, and days in hospital/ICU variables were compared by the Wilcoxon test; the other variables were compared by a chi-square test.

^†^ Logistic regression.

^‡^ According to previous surgical interventions declared in the medical history.

^§^ Percentage of patients with active antibiotic treatment at 8 hours of sepsis diagnosis. Antibiotics were categorized in the following classes: penicillins, cephalosporins, fluoroquinolones, macrolides, aminoglycosides, carbapenems, β-lactamase inhibitors, lipopeptides, nitroimidazoles, and oxazolidinones.

^‖^ Two or more affected organs based on SOFA scores.

^¶^ Presence of comorbidities (personal history): cancer, chronic diseases, diabetes, liver diseases, kidney diseases, previous severe infections, cardiac valve diseases.

Sequencing data yield an average of more than 32 million reads per sample (range of 10–70 million reads) (*see* Table E1 in the data supplement). After human DNA sequence removal, between 0.25% and 12.8% of the reads per sample identified as nonhuman were kept for downstream analyses. At this stage, we observed that a higher proportion of nonhuman reads in the sample was associated with ICU mortality in the patients (odds ratio [OR], 1.37; 95% confidence interval [CI], 1.03–2.21; *P* = 0.035). Compared with our previous findings (4), on average, 16S rRNA gene sequencing detected approximately two more genera per sample than shotgun sequencing at 1% abundance (mean difference, 2.04; 95% CI, −2.48 to 6.56). After metagenomic assembly and genome classification of the nonhuman reads, 14 genomes with an estimated completeness of 80–97% and low contamination were assembled across 12 patient samples (8 survivors, 4 deceased). All these genomes were classified as *Escherichia coli*, *Klebsiella pneumoniae*, *Pseudomonas aeruginosa*, *Acinetobacter baumannii*, *Achromobacter xylosoxidans*, *Staphylococcus aureus*, and *Haemophilus influenzae*, which correspond to the most abundant taxa detected at read level by MetaPhlAn4 in the samples.

**Figure 1.**
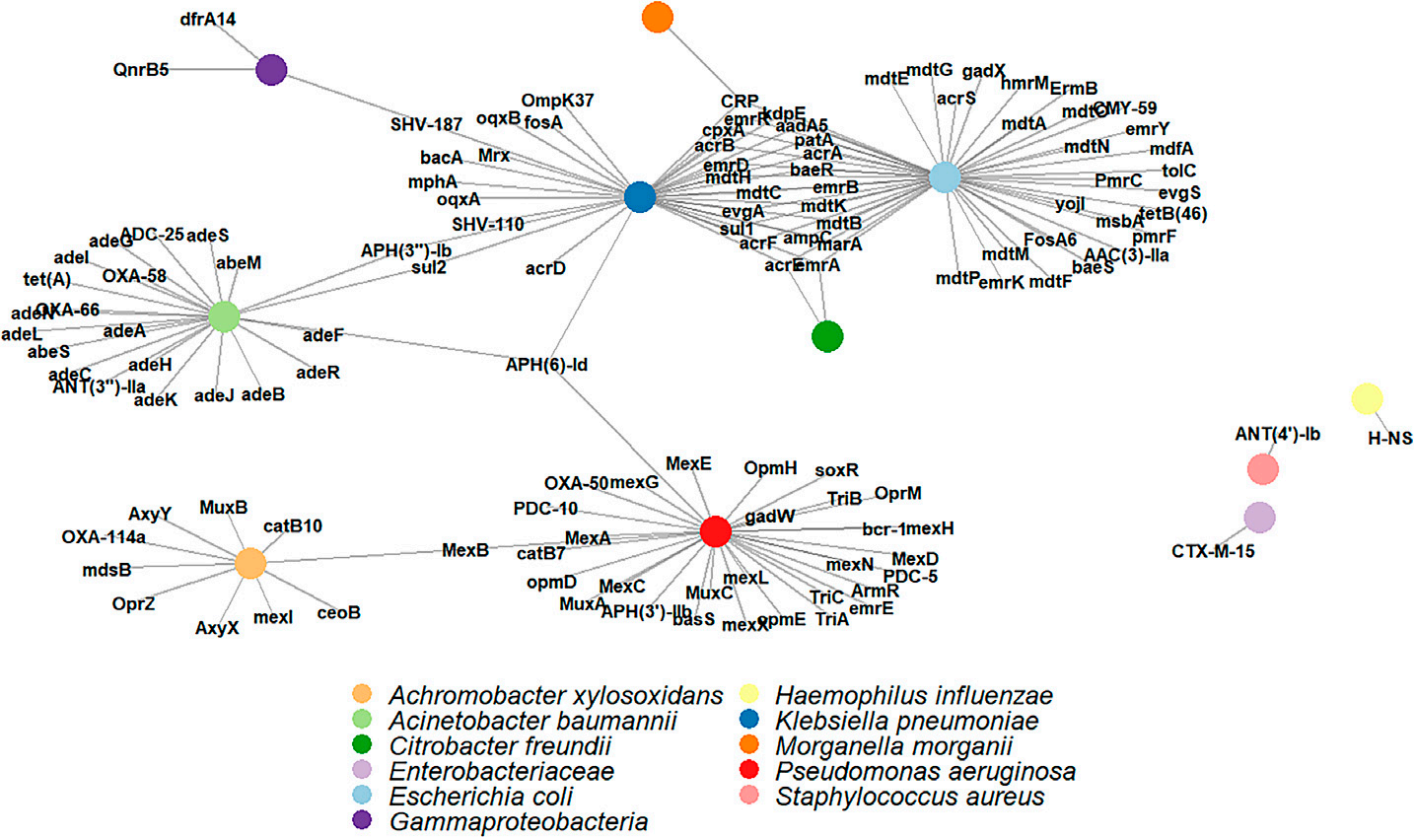
Network depicting the relationships between the antimicrobial resistances (AMR) and the bacterial taxonomy based on the contigs assembled in the AMR-positive patients.

ARGs were detected only in nine patients (127 ARGs in total), four of whom died in the ICU (Figures E1 and E2). We observed that the capacity to detect AMRs was associated with the proportion of nonhuman reads that were recovered from the samples (OR, 11.02; 95% CI, 1.94–144.58; *P* = 0.023). The Comprehensive Antibiotic Resistance Database ontology and bin classifications indicated that the most frequent AMR findings were linked to aminoglycosides and β-lactam antibiotics. These were identified, almost entirely, in the assembled genomes from *E. coli* (*n* = 48), *K. pneumoniae* (*n* = 35), and *P. aeruginosa* (*n* = 31) (Figure 1). Finally, we assessed if the abundance of AMRs was associated with ICU mortality in this cohort of patients. We found that the number of AMRs was significantly higher (OR, 1.19; 95% CI, 1.03–1.40; *P* = 0.022) in deceased patients (57%; 95% CI, 18–90%) compared with surviving patients (20%; 95% CI: 7–40%). These results were robust to model adjustments for relevant demographic and clinical covariates or the antibiotic treatments in the sensitivity analysis (Table 1).

Our analysis revealed that the abundance of AMRs in the lungs within the first hours of sepsis diagnosis correlated with ICU mortality rates, providing a qualitative survey of bacterial coinfections likely contributing to sepsis severity in these patients and making it the first study linking lung microbiome AMRs to ICU mortality in patients with sepsis. A possible explanation for this is that patients at higher risk of death are admitted with or develop in their lungs a higher load of AMRs (e.g., due to nosocomial exposures or iatrogenic effects of ICU interventions), which may hamper infection control during antibiotic treatment.

Predominant gram-negative bacteria like *E. coli*, *P. aeruginosa*, and *K. pneumonia* harboring diverse resistance mechanisms have been previously identified as common sepsis-causing pathogens (6). A significant proportion of the detected resistance determinants in these species, as well as in most of the AMR-positive samples from our study, belong to the aminoglycoside, β-lactam, and peptide antibiotic classes, which have been linked to a substantial impact on the efficacy of antimicrobial treatment (7, 8). Independent studies have found that poor outcomes in mechanically ventilated critically ill patients were associated with increased bacterial burden in the lung microbiome (9). This agrees with our observation, in which a higher proportion of nonhuman reads were associated with ICU mortality in our patient series. Taken together, our results emphasize the significance of understanding bacterial pathogens in sepsis severity and early response strategies.

This work has a series of limitations, which include a small sample size from a single ICU, preventing optimal confounding effect assessments, and the study’s focus on bacterial reads because of the prevalence of bacterial sepsis, which restricts conclusions regarding nonbacterial pathogens (10). In addition, we did not have the possibility to seek validation of results in independent larger studies or access to patient samples before the onset of sepsis to serve as controls. Finally, the results may be affected by the technical challenges in identifying AMRs because of the small proportion of nonhuman reads recovered for some samples. Despite the advancements in high-throughput sequencing, metagenomic data and antibiotic resistance profiles of lung samples are underrepresented in the literature compared with the gut or oral microbiome. As in chronic diseases (11), we suggest that future metagenomic applications could potentially enhance early-response strategies to sepsis, anticipating a growing relevance in clinical applications.
